# 

**Published:** 1888-02-11

**Authors:** 


					PRICE ONE PENNY.
72, VoL III-
February 11th, 1888.
I Registered at the General Post Office
as a Newspaper.]
Per Annum, 6s. 6d? post ft ee.
Monthly Parts, 6d.; post free, 7id.
Ditto per Ann.. 7s. ed. post free.
PA
WXk
? sitsrfi'^^i^aa'ifnini; [
"B arwaSHiLJs'"'?mTrl
ait l!fl!!ny>Wiil * limUM wi'l'
'^WvvpiUl
A Weekly Institutional Journal of
Science, flfoebtcme, anb lp>bUantbrop?.

				

